# Preventing subclinical necrotic enteritis through *Lactobacillus johnsonii* BS15 by ameliorating lipid metabolism and intestinal microflora in broiler chickens

**DOI:** 10.1186/s13568-017-0439-5

**Published:** 2017-06-26

**Authors:** Xiaodan Qing, Dong Zeng, Hesong Wang, Xueqin Ni, Lei Liu, Jing Lai, Abdul Khalique, Kangcheng Pan, Bo Jing

**Affiliations:** 1 Animal Microecology Institute, College of Veterinary, Sichuan Agricultural University, Chengdu, Sichuan China; 2 Key Laboratory of Animal Disease and Human Health of Sichuan Province, Chengdu, Sichuan China

**Keywords:** *Lactobacillus johnsonii*, Subclinical necrotic enteritis, Lipid metabolism, Intestinal flora, Q-RNA

## Abstract

Increasing studies have focused on the beneficial effects of *Lactobacillus johnsonii* in certain diseases. Here, we studied the prevention ability of a probiotic strain, *L. johnsonii* BS15 on subclinical necrotic enteritis (SNE), and its underlying mechanism. 180 male Cobb 500 chicks were randomly allotted into three groups and administrated with BS15 (1 × 10^6^ cfu/g) or Man Rogosa Sharpe liquid medium throughout a 28-day experimental period. With the exception of the normal group, SNE infection was treated for the remaining experimental period after the chicks were fed with normal diet 14 days. Results showed that BS15 notably suppressed the SNE-induced loss of average daily gain and liver functional abnormality. Additionally, BS15 facilitated lipid metabolism of SNE boilers when the contents of peroxisome proliferator activated receptor γ and adipose triglyceride lipase in adipose tissue and serum high-density lipoprotein cholesterol decreased. BS15 also attenuated the hepatic lipid accumulation of stricken chicks by suppressing the genes expression of acetyl-CoA carboxylase, fatty acid synthase and sterol regulatory element binding protein-1c as well as stimulating the genes expression of peroxisome proliferator activated receptor α and carnitine palmitoyltransferase-1. Moreover, BS15 enhanced the development of SNE gut by improving the intestinal development and digestion as well as adjusting the gut microflora. Therefore, BS15 may provide a promising natural preventative strategy against SNE, which may be contributed to the amelioration of lipid metabolism and intestinal microflora.

## Introduction

Subclinical necrotic enteritis (SNE), caused by enterotoxigenic *Clostridium perfringens*, is a major cause of human foodborne disease, and currently, most outbreaks of SNE are related with meat products and poultry (Cooper et al. 2013). The incidence of SNE is on the rise in recent years, which is firsthand associated with the ban of the European Union on using subtherapeutic antibiotics as feed additives in poultry breeding (Tsiouris 2016), finding alternatives to replace antibiotics has gained increasing concerns (Fallah et al. 2013). So far, numerous studies established that probiotics are one of the best ideal alternatives for antibiotic growth promoters in poultry (Makawy 2014; Zhuang et al. 2015; Kim et al. 2016). *Lactobacillus* strains, one of the most widely used probiotics in poultry industry, were supposed to ameliorate the intestinal flora homeostasis (Chiva et al. 2002; Wang and Huang 2014), improve the growth performance (Lee et al. 2013; Suda et al. 2014) and prevent some fowl diseases (Layton et al. 2013; Saintcyr et al. 2017). Moreover, recent reports highlighted the link between liver and gut on the structure and function, particularly the role of lipid metabolism (Compare et al. 2012; Endo et al. 2013), and the liver of poultry is a vital organ where the majority of the de novo fatty acid synthesis process usually occurs. The composition of intestinal flora influenced by probiotics plays a pivotal role in preventing liver diseases (Chávez-Tapia et al. 2015), and chickens are susceptible to multifarious pressures factors in lipid metabolism (Saneyasu et al. 2013). These studies strongly imply the importance of lipid metabolism in the relationship between liver and intestinal flora. Our previous study found that the lipid metabolism of broilers was disturbed by necrotic enteritis, but the addition of *Bacillus licheniformis* in the basal diet alleviated this damage (Zhou et al. 2016), proving that probiotics can promote the growth of animals via improving the lipid metabolism. However, little is known about the lipid metabolism in SNE infection chickens caused by *C. perfringens*, furthermore, the mechanism of *Lactobacillus johnsonii* is still unclear as probiotics to prevent SNE.

*Lactobacillus johnsonii* BS15 (CCTCC M2013663) strain as probiotics can prevent non-alcoholic fatty liver disease by attenuating inflammation and mitochondrial injury, and improving the gut environment in obese mice (Xin et al. 2014), it was isolated from homemade yogurt collected from the Hongyuan Prairie, Aba Autonomous Prefecture, China. We hypothesized that *L. johnsonii* BS15 accretion may relieve the injury caused by SNE infection. In the present study, we undertaken to investigate whether there are improvement effects of BS15 on growth performance, the development and digestive ability of intestine, serum lipid, genes expression of adipose tissue and liver enzymes related to lipid metabolism, and intestinal microbiota homeostasis in SNE broilers.

## Materials and methods

### Strains

The amounts of *L. johnsonii* BS15 (CCTCC M2013663) cells were confirmed by heterotrophic plate counts after culturing in MRS broth at 37 °C for 36 h under anaerobic environment. And then cells were collected, washed with saline, and suspended in phosphate buffered saline (pH 7.0) containing 1 × 10^9^ colony-forming unit (cfu)/ml. The culture was mixed with a basic ration at a level of 1 g/kg (0.1%, m/m) per day to guarantee the viability of bacteria cells throughout the experimental stage.

A NetB toxin positive type A *C. Perfringens* (CVCC2030) strain was originated from the intestine of a broiler with clinically diagnosed with necrotic enteritis was obtained from China Veterinary Culture Collection Center. Before inoculation of the chickens, the bacteria were cultured in a cooked meat medium for 24 h at 37 °C in the anaerobic cabinet. The strain was aseptically stored in fluid thioglycollate medium overnight at the same environment.

### Animals and treatment

A total of 180 one-day-old male chicks (Cobb 500) with similar body masses were obtained from Chia Tai broiler hatchery, Chengdu, China, and were fed on normal diet for 1 week to stabilize all metabolic condition. The diets were formulated consulting the NRC (1994) and shown in Table 1. Chicks were randomly allotted to three groups consisting of six replicates with ten chicks per replicate (each replicate was a pen) and housed in a humidity controlled room throughout a 28-day experimental period. The temperature was maintained 33 °C in the first week and then gradually decreased by 3 °C a week until it reached 24 °C. Lighting was provided 24 h/day. The three groups of chicks were fed as follows: normal control (NC, basal diet), SNE control (SNE, SNE experimental model), BS15 preventative group (BS15, basal diet + 1 × 10^6^ cfu BS15/g as feed + SNE experimental model). Feed and water were provided ad libitum and all animal experiments were performed under the guidelines approved by the Institutional Ethical Committee for the care and use of laboratory animals.

**Table 1:** Composition of the basal diets for broilers

Ingredient^a^	Diet (%)
Ground yellow corn	56.0
Soybean meal	37.0
Soybean oil	3.66
Ground limestone	0.57
Dicalcium phosphate	1.80
Salt	0.30
Choline chloride	0.10
DL-Met	0.24
Micronutrients^b^	0.33
Calculated nutrients level (%)	
ME (MJ kg^−1^)	12.39
CP	21.17
Lys	1.19
Met	0.50
Met + Cys	0.86
Ca	0.85
Nonphytate P	0.44

^a^ Ingredient and nutrient composition are reported on as-fed basis

^b^ Micronutrients are provided per kilogram of diet: vitamin A (all-trans retinol acetate), 12,500 IU; cholecalciferol, 2500 IU; vitamin E (all-rac-a-tocopherol acetate), 18.75 IU; vitamin K (menadione Na bisulfate), 5.0 mg; thiamin (thiamin mononitrate), 2.5 mg; riboflavin, 7.5 mg; vitamin B6, 5.0 mg; vitamin B12, 0.0025 mg; pantothenate, 15 mg; niacin, 50 mg; folic acid, 1.25 mg; biotin, 0.12 mg; Cu (CuSO_4_·5H_2_O), 10 mg; Mn (MnSO_4_·H_2_O), 100 mg; Zn (ZnSO_4_·7H_2_O), 100 mg; Fe (FeSO_4_·7H_2_O), 100 mg; I (KI), 0.4 mg; Se (Na_2_SeO_3_), 0.2 mg

At 15 days of age, chicks were gavaged with 20,000 *Eimeria acervulina* oocysts and 5000 *Eimeria maxima* oocysts per chick except for the NC group. Unchallenged control chicks received the same volume of sterile phosphate buffered saline instead of *Eimeria* oocysts. All *Eimeria oocysts* gavaged chicks were tolerated orally with 1 ml of *C. perfringens* (2.2 × 10^8^ cfu/ml) on days 18, 19, 20, 21 and 22. Meanwhile, unchallenged chicks accepted 1 ml of sterile fluid thioglycollate broth. BS15 group was supplemented with 1.0 × 10^6^ cfu BS15/g throughout the trial, and all samples were collected on day 28.

### Growth performance and sample collection

No death was recorded during the entire period of experiment. The weight and feed consumption of chicks were recorded on days 1 and 28, respectively, and average daily feed intake (ADFI) and average daily gain (ADG) were calculated from these values. Feed conversion rate (FCR) was calculated the ratio of ADFI to ADG. On day 28, 18 chicks from each group (one chick per pen) were randomly selected and humanely terminated. Abdominal fat percentage was then evaluated as the percentage of carcass weight by removing and weighting the abdominal fat pad surrounding the gizzard, cloaca, and adjacent muscles.

At the end of experiment, six chicks (one chick per pen) in each group were randomly selected to collect 5 ml of blood from the wing vein for separating the serum samples. And then chicks were euthanized under the institutional animal care guidelines. The liver and abdominal adipose tissue were quickly excised, weighted, and frozen in liquid nitrogen immediately before all samples were ultimately stored at −80 °C for succeeding use. Total liver and adipose tissue RNA were extracted using RNAiso Plus reagent (TaKaRa, Dalian, China) in the light of the specifications. RNA quality was checked by 2% (w/v) agarose gel electrophoresis, and the concentrations were analyzed by the Nano Drop spectrophotometer (Beckman Coulter DU 800, Fullerton, CA, USA). The extracted RNA were reverse transcribed into cDNA immediately using a Prime Script™ RT reagent kit (TaKaRa, Dalian, China) in accordance with the procedures manual of the manufacturer. Eventually, all cDNA were stored at −30 °C to be used as templates for quantitative real-time polymerase chain reaction (qRT-PCR). Fresh digesta and intestinal segment (jejunum and ileum) of chicks were gathered, and temporary stored in liquid nitrogen promptly before sending to the laboratory where they were preserved at −80 °C. Total isolated DNA from the jejunal and ileal content were extracted by the E.Z.N.A.™ stool DNA isolation kit (Omega Bio-Tek, Doraville, CA, USA) basing on the protocol. The concentration of final elution was then performed by a spectrophotometer (Beckman Coulter DU 800, Fullerton, CA, USA). Qualitative analyses of extracted DNA were evaluated from 2% (w/v) agarose gel electrophoresis, and all the DNA products were deposited at −30 °C for further analysis.

### Determination of serum biochemical values

The concentration of alanine aminotransferase (ALT) and aspartate transaminase (AST) in serum were assayed with the commercial kits obtained from Nanjing Jiancheng Bioengineering Institute (Nanjing, Jiangsu, China). The contents of triglyceride (TG), total cholesterol (TC), high-density lipoprotein cholesterol (HDL-C) and low-density lipoprotein cholesterol (LDL-C) in serum samples were determined by a GS200 Automatic Biochemical Analyzer (Shenzhen Genius Electronics Co., Ltd., Shenzhen, China) following the instructions of the corresponding reagent kit.

### Determination of development and digestive enzyme activities in intestines

Epidermal growth factor (EGF) and insulin-like growth factors-1 (IGF-1) in the intestinal content were quantified using the ELISA kits specific for chick (RD Ltd, USA). The levels of these indices were detected by the standard curve and expressed as nanogram per milliliter (ng/ml). The activity of amylase, trypsin and lipase were tested using commercially available kits (Nanjing Jiancheng Bioengineering Institute) in accordance with the instructions as indices for intestinal digestion capacities.

### Quantitative real time PCR (qRT-PCR) analysis of genes expression

qRT-PCR analysis was performed by using a CFX96 Real-Time system (Bio-Rad, Hercules, CA, USA) with SYBR^®^ Premix Ex Taq™ II (TaKaRa, Dalian, China). The protocol was implemented predenaturation for 3 min at 95 °C, followed by 40 cycles of 15 s denaturation at 94 °C and 30 s annealing at suitable temperatures, extension at 72 °C for 30 s, and the melting curve analysis was conducted to verify the purity of the PCR products. Primer’s information is shown in Table 2. The abundance of relative gene expression was presented as fold changes after normalization to the glyceraldehyde-3-phosphate dehydrogenase (GADPH), which was used as the eukaryotic housekeeping gene, and finally the data were analyzed by using Microsoft Excel software with 2^−ΔΔCt^ relative quantitative method. All samples (n = 6) per group were done in triplicate, and the average values of these arrangements were used to evaluate the mRNA expression.

**Table 2:** Gene-specific primers of the lipid metabolism related enzyme

Gene name	Primer sequence (5 → 3)	Tm (°C)/size (bp)	Accession
ACC	F:AATGGCAGCTTTGGAGGTGT R:TCTGTTTGGGTGGGAGGTG	60.9/136	NM205505
FAS	F:CTATCGACACAGCCTGCTCCT R:CAGAATGTTGACCCCTCCTACC	62.0/107	J03860
SCD1	F:ACCATACATTCCCCTACGACT R:TTTTCCGGGCCAAGATGACC	56.0/144	NM204890
SREBP-1c	F:GAGGAAGGCCATCGAGTACA R:GGAAGACAAAGGCACAGAGG	60.3/220	AY029224
PPARα	F:TGGACGAATGCCAAGGTC R:GATTTCCTGCAGTAAAGGGTG	60.3/813	AF163809
CPT-1	F:CAATGAGGTACTCCCTGAAA R:CATTATTGGTCCACGCCCTC	57.5/337	AY675193
ACOX	F:ATGTCACGTTCACCCCATCC R:AGGTAGGAGACCATGCCAGT	54.0/133	NM001006205
PPARγ	F:CCAGCGACATCGACCAGTT R:GGTGATTTGTCTGTCGTCTTTCC	57.5/145	AF163811
ATGL	F:TCCTTCACCTTCAGCGTCCA R:AGTGTTGTCCTCCATCTGGTC	54.0/113	EU852334
LPL	F:CAGTGCAACTTCAACCATACCA R:AACCAGCCAGTCCACAACAA	60.0/150	NM205282
GADPH	F:GGTGAAAGTCGGAGTCAACGG R:CGATGAAGGGATCATTGATGGC	58.4/108	NM204305

*ACC* acetyl-CoA carboxylase, *FAS* fatty acid synthase, *SCD1* stearoyl-CoA desaturase-1, *SREBP-1c* sterol regulatory element binding protein-1c, *PPARα* peroxisome proliferator activated receptor α, *CPT-1* carnitine palmitoyltransferase-1, *ACOX* acyl CoA oxidase, *PPARγ* peroxisome proliferator activated receptor γ, *ATGL* adipose triglyceride lipase, *LPL* lipoprotein lipase, *GADPH* glyceraldehyde-3-phosphate dehydrogenase

### Quantitative PCR quantification of bacteria in jejunum and ileum

A CFX96 Real-Time system (Bio-Rad, CA, USA) and SYBR^®^ Premix Ex Taq™ II (TaKaRa, Dalian, China) were used to express quantitative PCR (qPCR), to estimate the abundance of bacteria in jejunum and ileum. The amplifying primers of the targeted microflora were showed in Table 3. All reactions were done in triplicate and in a 25 μl reaction mixture including 9.5 μl of sterile deionized water, 12.5 μl SYBR^®^ Premix Ex Taq™ II, 1 μl of forward, and reverse primer and 1 μl template DNA. The protocols were 1 cycle at 95 °C for 3 min, followed by 40 cycles at 95 °C for 15 s, annealing at the optimum temperatures for 30 s, 72 °C for 30 s and melting curves data were analyzed to monitor the purity of the PCR product.

**Table 3:** Primers used for the quantification of the specific bacterial divisions’ expression by real-time PCR

Target species	Primer sequence (5 → 3)	Annealing temp (°C)	Product size (bp)	Reference
Total bacteria	F: CGGYCCAGACTCCTACGGG R: TTACCGCGGCTGCTGGCAC	60.0	130	Guo et al. (2008)
*Firmicutes*	F: GGAGYATGTGGTTTAATTCGAAGCA R: AGCTGACGACAACCATGCAC	64.5	126	
*Bacteroidetes*	F: GGARCATGTGGTTTAATTCGATGAT R: AGCTGACGACAACCATGCAG	60.0	126	
*Lactobacillus* spp.	F: AGCAGTAGGGAATCTTCCA R: CACCGCTACACATGGAG	64.5	341	Walter et al. (2001)
*Enterococcus* spp.	F: CCCTTATTGTTAGTTGCCATCATT R: ACTCGTTGTACTTCCCATTGT	59.6	144	Rinttilä et al. (2004)
*Streptococcus* spp.	F: AGAGTTTGATCCTGGCTCAG R: GTTAGCCGTCCCTTTCTGG	56.8	485	Franks et al. (1998)
*Clostridium cluster I*	F: ATGCAAGTCGAGCGAKG R: TATGCGGTATTAATCTYCCTTT	60.0	120	Rinttilä et al. (2004)
*Clostridium cluster IV*	F: GCACAAGCAGTGGAGT R: CTTCCTCCGTTTTGTCAA	60.0	230	Matsuki et al. (2004)
*Clostridium cluster XIVa*	F: AAATGACGGTACCTGACTAA R: CTTTGAGTTTCATTCTTGCGAA	60.0	440	Matsuki et al. (2002)
*Bifidobacterium*	F: TCGCGTCYGGTGTGAAAG R: CCACATCCAGCRTCCAC	56.9	243	Rinttilä et al. (2004)
*Enterobacteriaceae*	F: CATTGACGTTACCCGCAGAAGAAGC R: CTCTACGAGACTCAAGCTTGC	62.5	195	Bartosch et al. (2004)

For the standard curves, the amplified products were cloned into the pMD19-T (TaKaRa) vector, and then transformed into DH5α (TianGen, Beijing, China) for amplification, and sequenced subsequently as described above. Positive clones were then extracted the plasmids using A E.Z.N.A.™ plasmid mini kit (Omega Bio-Tek, USA), and the quantitative analysis of plasmid DNA were evaluated by the Nano Drop spectrophotometer (Beckman Coulter DU 800). The standard curves were created using triplicate tenfold serial dilutions of plasmid DNA. Copy numbers of the target microbiota for samples were calculated according to the standard curves.

### Data analysis

Data were expressed as mean ± SD and analyzed using one-way analysis of variance (ANOVA). Duncan’s multiple-range test was used for multiple comparisons when a significant interaction was detected. All the statistical analyses were conducted using the SigmaPlot for Social Sciences version 13. P < 0.05 was considered statistically significant differences.

## Results

### Effect of BS15 on the growth performance of SNE broilers

The mean values of ADFI, ADG and FCR are shown in Table 4. ADFI did not vary significantly among all groups throughout the experimental period. However, the BS15 supplementation suppressed the decrease in ADG (*P* < 0.05) and the increase in FCR (*P* > 0.05), which both caused by SNE.

**Table 4:** Effects of *Lactobacillus johnsonii* BS15 on growth performance

Parameter	NC	SNE	BS15	P
ADFI, g/day	60.71 ± 2.29	60.60 ± 2.33	61.10 ± 3.13	0.944
ADG, g/day	39.24 ± 2.23^a^	35.82 ± 1.50^b^	38.54 ± 2.07^a^	0.020
FCR	1.55 ± 0.10^b^	1.69 ± 0.09^a^	1.59 ± 0.09^ab^	0.046

Means in the same row with no superscripts or with a common superscript letter do not differ significantly (P < 0.05). Data are presented with the mean ± standard deviation (n = 6; pen was used as the experimental unit). NC = basal diet without supplementation; SNE = subclinical necrotic enteritis experimental model group; BS15 = basal diet with 1.0 × 10^6^ cfu BS15/g diet as feed

*ADFI* average daily feed intake, *ADG* average daily gain, *FCR* feed conversion rate (*ADFI*/*ADG*)

### Effect of BS15 on the serum biochemical parameters

As shown in Table 5, the concentration of ALT and AST in serum were both significantly increased in the SNE group compared with the NC group (*P* < 0.05), and the BS15 supplementation reduced the levels of ALT (*P* < 0.05) and AST (*P* > 0.05) with SNE infection. Additionally, the weak chicks in the BS15 group displayed lower TC level (*P* > 0.05) and higher HDL-C level (*P* < 0.05) in serum compared with those in the SNE group. The amounts of TG and LDL-C in serum were no significantly influence with any treatments (*P* > 0.05), but the mean values of parameters described above in the BS15 group did not differ significantly from those of the negative group.

**Table 5:** Effects of *Lactobacillus johnsonii* BS15 on serum biochemical parameters

Parameter	NC	SNE	BS15	P
ALT, IU/l	11.58 ± 1.07^b^	13.87 ± 1.39^a^	12.02 ± 1.20^b^	0.013
AST, IU/l	131.70 ± 13.23^b^	155.21 ± 16.79^a^	142.23 ± 14.49a^b^	0.048
TG, mmol/l	0.29 ± 0.03	0.25 ± 0.03	0.27 ± 0.03	0.108
TC, mmol/l	2.52 ± 0.23^b^	2.92 ± 0.17^a^	2.71 ± 0.26^ab^	0.026
HDL-C, mmol/l	2.91 ± 0.31^a^	2.40 ± 0.29^b^	2.79 ± 0.26^a^	0.020
LDL-C, mmol/l	1.01 ± 0.16	1.24 ± 0.17	1.12 ± 0.16	0.086

Means in the same row with no superscripts or with a common superscript letter do not differ significantly (P < 0.05). Data are presented with the mean ± standard deviation (for six replicates of one chick per cage). NC = basal diet without supplementation; SNE = necrotic enteritis experimental model group; BS15 = basal diet with 1.0 × 10^6^ cfu BS15/g diet as feed

*AST* aspartate aminotransferase, *ALT* alanine aminotransferase, *TG* triglycerides, *TC* total cholesterol, HDL-C high-density lipoprotein cholesterol, LDL-C low-density lipoprotein cholesterol

### Effect of BS15 on the development and digestion of intestine

**Fig. 1:**
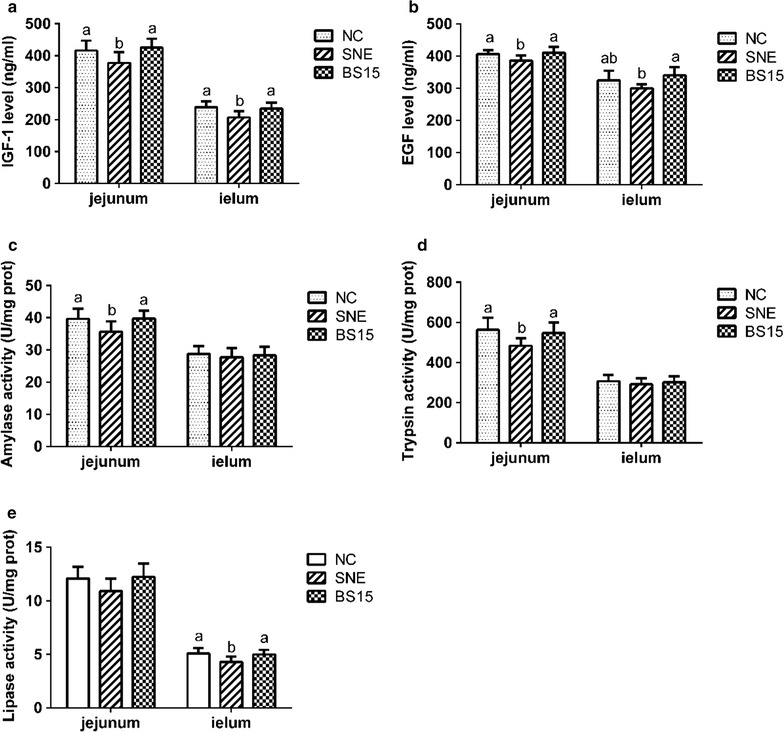
Effects of *Lactobacillus johnsonii* BS15 on the development and digestion of intestine. Means in the same row with no superscripts or with a common superscript letter do not differ significantly (P < 0.05). Data are presented with the mean ± standard deviation (for six replicates of one chick per cage). **a**, **b** The IGF-1 and EGF levels in jejunum and ileum. **c**–**e** The activities of amylase, trypsin and lipase in jejunum and ileum

Figure 1 shows that probiotic had significant positive effect on the level of IGF-1 and EGF in jejunum and ileum of the stricken chicks (*P* < 0.05), and there are no significant difference shown between the BS15 group and the normal group. Moreover, BS15 supplement significantly suppressed (*P* < 0.05) the decrease in the activities of amylase and trypsin in jejunum and the level of lipase in ileum, which both caused by SNE infection. The levels of lipase in jejunum as well as amylase and trypsin in ileum were unaffected by BS15 treated or SNE infected. Meanwhile, there is no obviously different between the BS15 group and the control about above digestive enzymes.

**Fig. 2:**
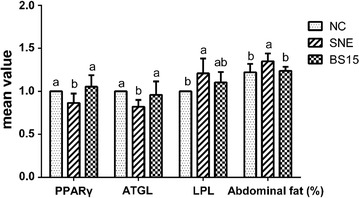
Effects of *Lactobacillus johnsonii* BS15 on abdominal fat percentage and abdominal fat metabolism related mRNA expression in broiler. Means in the same row with no superscripts or with a common superscript letter do not differ significantly (P < 0.05). Data are presented with the mean ± standard deviation (n = 6; pen was used as the experimental unit). *PPAR*γ peroxisome proliferator activated receptor γ, *ATGL* adipose triglyceride lipase, *LPL* lipoprotein lipase

### Fat deposit and genes expression of lipid metabolism related enzyme in adipose tissue

As shown in Fig. 2, BS15 addition exhibited the remarkable decrease (*P* < 0.05) in the rate of abdominal fat, compared with the SNE group. The lower expressions of PPARγ and ATGL as well as the higher expression of lipoprotein lipase (LPL) were revealed in SNE group than that in the health chicks (*P* < 0.05). Besides, the genes expression of PPARγ and ATGL were increased in BS15 group compared with the SNE group. There was no obviously alterations of LPL between the BS15 prevention and the SNE group, although the BS15 group displayed a lower expression.

### Effect of BS15 on genes expression of enzymes related with lipid metabolism in liver

**Fig. 3:**
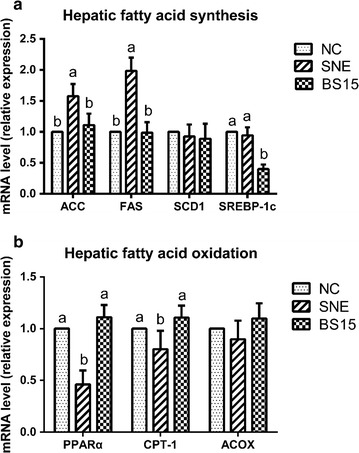
The relative mRNA expressions of lipid metabolism related enzymes in liver. Means in the same row with no superscripts or with a common superscript letter do not differ significantly (P < 0.05). Data are presented with the mean ± standard deviation (for six replicates of one chick per cage). **a**, **b** The relative expression of *ACC*, *FAS*, *SCD*1, *SREBP*-1c, *PPAR*α, *CPT*-1 and *ACOX*, respectively

The consequence of BS15 supplementation on lipid metabolism were studied by analyzing the expressions of several genes involved in the regulation of hepatic fatty acid synthesis (ACC, FAS, SCD1, SREBP-1c) and hepatic fatty acid oxidation (PPARα, CPT-1, ACOX). Figure 3 shows the significantly downregulations of PPARα and CPT-1 as well as the significantly upregulations of ACC and FAS in the SNE group compared with the NC group (*P* < 0.05). The treatment of BS15 significantly downregulated the hepatic expressions of ACC, FAS and SREBP-1c, as well as induced an obviously increase in the hepatic expressions of PPARα and CPT-1 compared with the SNE group (*P* < 0.05).

### Effect of BS15 on derangement of gut flora

Table 6 shows that the abundance of total bacteria, *Firmicutes*, *Enterococcus* spp., and *Bifidobacterium* did not vary significantly among three groups whether in jejunum or ileum, and the counts of *Clostridium cluster IV* and *Enterobacteriaceae* were also no considerable alteration among all groups in jejunum. Whereas the abundance of *Streptococcus* spp. in jejunum and ileum, and *C. cluster I* in jejunum, as well as *Enterobacteriaceae* in ileum were remarkably enhanced (*P* < 0.05); the abundance of *Bacteroidetes*, *Lactobacillus* spp. and *C. cluster XIVa* in jejunum and ileum, and *C. cluster IV* in ileum were significantly reduced (*P* < 0.05) upon treatment with SNE infection without BS15 as compared with the normal chicks. Interestingly, after BS15 treatment, the abundance of *Lactobacillus* spp. and *C. cluster XIVa* in jejunum, and *Bacteroidetes*, *Lactobacillus* spp. and *C. cluster IV* in ileum remained at higher level, whereas that of *Streptococcus* spp. in jejunum and ileum were markedly lower than those in the SNE group (*P* < 0.05).

**Table 6:** Effect of *Lactobacillus johnsonii* BS15 on the gut microbiota

Parameter	NC	SNE	BS15	P
Jejunum				
All bacteria	9.64 ± 0.94	9.72 ± 0.87	9.82 ± 0.72	0.937
*Firmicutes*	8.83 ± 1.16	8.91 ± 0.96	9.26 ± 0.80	0.729
*Bacteroidetes*	7.73 ± 0.71^b^	6.41 ± 0.95^b^	7.00 ± 0.81^ab^	0.047
*Lactobacillus* spp.	8.43 ± 0.62^ab^	7.76 ± 0.77^b^	8.89 ± 0.63^a^	0.035
*Enterococcus* spp.	8.25 ± 0.84	8.00 ± 1.25	8.27 ± 0.61	0.863
*Streptococcus* spp.	6.75 ± 0.68^b^	7.61 ± 0.41^a^	6.81 ± 0.58^b^	0.035
*Clostridium cluster I*	6.01 ± 0.79^b^	7.35 ± 0.74^a^	6.81 ± 0.68^ab^	0.021
*Clostridium cluster IV*	8.26 ± 1.02	7.14 ± 0.75	8.13 ± 1.05	0.116
*Clostridium cluster XIVa*	7.77 ± 0.96^a^	6.52 ± 0.72^b^	7.91 ± 0.74^a^	0.018
*Bifidobacterium*	7.76 ± 0.84	7.63±0.68	7.80±0.42	0.757
*Enterobacteriaceae*	8.80 ± 0.94	9.37 ± 1.17	8.75 ± 1.25	0.586
Ileum				
All bacteria	9.83 ± 0.90	9.85 ± 0.76	9.82 ± 0.83	0.998
*Firmicutes*	8.49 ± 0.70	8.05 ± 0.86	8.59 ± 0.92	0.512
*Bacteroidetes*	7.86 ± 0.99^a^	6.76 ± 0.57^b^	7.70 ± 0.63^a^	0.049
*Lactobacillus* spp.	8.26 ± 1.01^a^	7.11 ± 0.60^b^	8.36 ± 0.94^a^	0.045
*Enterococcus* spp.	8.20 ± 0.83	7.75 ± 0.85	7.99 ± 0.70	0.638
*Streptococcus* spp.	7.19 ± 0.73^ab^	7.93 ± 0.74^a^	6.89 ± 0.50^b^	0.044
*Clostridium cluster I*	5.75 ± 0.54^b^	6.28 ± 0.65^ab^	6.64 ± 0.49^a^	0.046
*Clostridium cluster IV*	7.54 ± 0.71^a^	6.26 ± 0.89^b^	7.77 ± 1.22^a^	0.034
*Clostridium cluster XIVa*	7.71 ± 0.39^a^	7.04 ± 0.40^b^	7.55 ± 0.47^ab^	0.035
*Bifidobacterium*	7.14 ± 0.82	6.83 ± 0.64	7.19 ± 0.76	0.680
*Enterobacteriaceae*	8.54 ± 0.30^b^	9.67 ± 0.67^a^	9.25 ± 1.03^ab^	0.049

Log_10_DNA gene copies of all bacteria, *Firmicutes*, *Bacteroidetes*, *Lactobacillus* spp., *Enterococcus* spp., *Streptococcus* spp., *Clostridium cluster I*, *Clostridium cluster IV*, *Clostridium cluster XIVa*, *Bifidobacterium*, *Enterobacteriaceae*, and in the jejunum, and ileum, respectively. Log_10_DNA gene copies quantification data were normalized to the standard curve lines and presented with the mean ± standard deviation (n = 6)

Superscript letters a, b, c, and d represent (P < 0.05) between the group and NC group, SNE group and BS15, respectively

## Discussion

As a potential probiotic strain, BS15 have been proved a wide variety of health benefits ranging from enhanced immunity to improved the lipid metabolism in previous study (Xin et al. 2014; Wang et al. 2017). The 3 weeks treatment of BS15 exhibited the beneficial prevention in SNE broilers through adjusting the intestinal flora which did an initial protection. The significant increase in feed conversion rate; the abdominal fat rate; and the serum biochemical indicators (ALT, AST, TC, HDL-C) in the SNE group indicated the success of SNE model that is vital for the process of this experiment. However, the preliminary supplementation of BS15 alleviated this disease.

In the current study, significantly reduced ADG and damaged FCR were concrete embodiment of the poor growth performance of SNE chicks as compare with the normal group. These results were consistent with former research that reported the poor growth in SNE chicks (Wang et al. 2017). However, the treatment of BS15 positively ameliorated SNE chicks’ poor growth by significantly increasing ADG, although there was no change of ADFI in these three groups from 1 to 28 day, indicating that BS15 as probiotics improved the animal’s growth (Zhou et al. 2010; Zhou et al. 2016). Moreover, BS15 not obviously affected FCR which mirrors the intestinal digestibility of nutrients (Afrouziyeh et al. 2014), but a tendency that the value of FCR in the BS15 group was close to the normal group and below in the PC group was found. Maybe the not enough feeding time and relatively serious chronic damage in intestine of the stricken chicks both inhibited the probiotic function of BS15.

Numerous researches shown that ALT and AST are key enzymes measured in serum or plasma to investigate liver diseases (Carobene et al. 2013), and the ratio of AST to ALT can be used to identify non-alcoholic fatty liver disease (Goh et al. 2015). The activities of ALT and AST in the serum can also observed for the indication of hepatic dysfunction (Chen et al. 2015; Liang et al. 2015). In present study, the higher levels of ALT and AST were observed in serum of SNE-infected chicks compared with the negative chickens, revealing the success of SNE model by the coinfection of *C. perfringens* and *Eimeria*. The supplementation of BS15 in diet not markedly improved AST, but notably decreased the level of ALT in sick chickens, suggesting that BS15 as probiotics maybe plays a positive role in alleviating liver injury (Xin et al. 2014). Besides, biological activities of serum lipoproteins are associated with blood lipid metabolism (Jansen 2014). Li et al. (2011) stated that polysorbates can reduce serum total cholesterol and low-density lipoprotein levels in hyperlipidemic mice and rats. Yang et al. (2014) indicated that improving the serum lipid profile, i.e., triglyceride and total cholesterol, is the most effective at decreasing liver lipid accumulation. These similar trends were observed in our study, which shown that the greater increase of HDL-C in SNE broilers after BS15 treatment, although there was no significant declination of TC and LDL-C in the BS15 group compared with the SNE group, and the TG was found same among all groups. These results suggest that the BS15 supplementation can help to remit dyslipidemia caused by SNE.

IGF-1 is found as a crucial regulator to command developmental bone growth and energy metabolism (Sheng et al. 2014), and improve small intestine enzymes activity in broilers (Moosavinasab et al. 2015). EGF, as a polypeptide growth factor, has a close relationship with intestinal growth regarding promoting cell proliferation (Chan et al. 2015) and the restoration of damaged epithelium (Liu et al. 2016a). Xu et al. (2015) demonstrated that EGF maintains the structural integrity and function of the small intestine in early-weaned piglets. In this data, the levels of IGF-1 in jejunum and ileum and the level of EGF in jejunum in the SNE-infected chicks were notably lower than that of the negative group, implying the poor intestinal development in SNE chicks. Whereas BS15 addition significantly enhanced the contents of IGF-1 and EGF both in the jejunum and ileum on the basis of the infected chicks, suggesting the recovery from injury of intestinal segments. Besides that, the similar consequence that probiotics improve the intestinal digestion by raising the activities of various intestinal digestive enzymes (Vand et al. 2014; Shori 2015) was also proved in this present study, and showing that the poor growth of SNE broilers were improved by BS15 through enhancing intestinal digestion ability, which was consistent with the previous result that FCR in BS15 group was lower than that in SNE group.

Abdominal fat of broilers is closely related with adipose deposition and always considered to be an added waste for treating effluent from processing (Fouad and Elsenousey 2014). In data of current study, the abdominal fat in BS15 group was markedly reduced when compared with SNE group, indicating that BS15 supplementation relieved abdominal adipose deposition by SNE infection. This is in accordance with previous conclusions that BS15 suppressed the fat deposition (Liu et al. 2016b; Wang et al. 2017) and improved the obesity caused by nonalcoholic fatty liver disease in mice (Xin et al. 2014). Adipocyte differentiation is regulated by various transcription factors, especially PPARs (Lefterova et al. 2014). PPARγ as a member of PPARs is mainly rich in adipose tissue, mediating adipocyte differentiation and maturation (Mayoral et al. 2015), and the overexpression of PPARγ significantly attenuated lipid accumulation (Yu et al. 2015). ATGL is another adipose decompose enzyme after hormone-sensitive lipase (HSL) found, playing an important regulation role in triglyceride accumulation (Heckmann et al. 2016). The increased amount of adipose tissue and TG accumulation in multiple tissue after ATGL gene knocked out in mice, revealing the key role of ATGL in steatolysis (Haemmerle et al. 2006). In the present study, the expressions of PPARγ and ATGL in adipose tissue were significantly upregulated in the BS15 group compared with the SNE group, which were supported by the previous verdicts that the lower abdominal fat in BS15 broilers than that in stricken chicks, although there was no obvious change of TG in serum. Researches proved that proper regulation of LPL is important for controlling the delivery of lipid nutrients to tissues (Davies et al. 2012), and it plays a dominant role in the hydrolysis of TG (Kanaki and Kardassis 2017). The consequence in our study shown that the gene expression of LPL was downregulated in SNE-infected chicks with replenishing BS15, but not significantly, implying that BS15 could effectively inhibit the abdominal fat deposit.

The hepatic genes expression that can regulate the ability of enzymes related with metabolic pathways, has always been received widespread attention. Inasmuch as unlike mammals, the liver of poultry is a vital organ where the majority of the de novo fatty acid synthesis process usually occurs. Except for SCD1 and ACOX, all the examined gene expressions of lipid metabolism related enzymes were changed by BS15 supplementation under the SNE infection in this study, suggesting that BS15 can ameliorate both the fatty acid synthesis and oxidation in the liver. SREBP-1c is a conserved transcription factor of the basic helix-loop-helix leucine zipper family that primarily regulates lipogenic enzymes and directly influences the expression of FAS, ACC and SCD1 (Ha et al. 2016). FAS and ACC are the key enzymes of chain fatty acid synthesis and extension (Tanos et al. 2012), and SCD1 is the rate-limiting enzyme that catalyzes the biosynthesis of saturated to monounsaturated fatty acids (Poudyal and Brown 2011). Although the expression of SCD1 showed no apparent response, in the current SNE model, BS15 accretion led to obviously inhibition of the expression of SREBP-1c and its target genes, ACC and FAS. This phenomenon may be attributed to the indubitable inhibition of BS15 on hepatic fatty acid synthesis in SNE chicks. In addition, PPARα predominantly expressed in liver has an intimate association with lipid catabolism in the PPARs, and can promote the hepatic genes expression of the fatty acid β-oxidation related key enzymes, such as CPT-1 and ACOX, which are implemented from mitochondria and peroxisomal respectively (Mandrup and Lane 1997; Song et al. 2010). In present study, higher expression of lipolytic genes, involving PPARα and CPT-1, were observed in the BS15 supplemented group compared with the SNE group, suggesting that BS15 addition may improve the suppression of the fatty acid β-oxidation in the SNE group.

The composition and quantity of intestinal flora closely related to the health and growth of the host (Sun et al. 2016). The results of current study shown that the rise of some beneficial bacterium and the decline of the harmful bacterium in probiotics group, suggesting that the intestinal flora structure of the stricken broiler was improved by *L. johnsonii* BS15. Recent literatures have shown that the growth performance of the host can be affected by the intake of exogenous probiotics through changing the gastrointestinal tract flora (Layton et al. 2013; Sánchez et al. 2015). *Bacteroidetes* is one of the main predominant flora in the GIT of chicken (Lan et al. 2005), which is also considered as the main bacteria to promote the use of carbohydrates (Brown et al. 2012). The proportion of *Firmicutes*/*Bacteroidetes* can be effective against obesity (Ley et al. 2006; Xin et al. 2014), which is a key indicator in response to the lipid metabolism (Cui et al. 2013). In the present study, the apparent reduction of *Bacteroidetes* in the jejunum and ileum of injured chicks in SNE group was observed, and the number of *Firmicutes* did not vary across all of the groups throughout the experimental period, implying the increase of the *Firmicutes*/*Bacteroidetes* ratio in SNE chicks. However, this trend was attenuated by supplementation of probiotics, manifesting that the *L. johnsonii* BS15 addition may play a positive effect on lipid metabolism via adjusting the ratio of *Firmicutes*/*Bacteroidetes* in the gut to decline the fat deposition.

The use of probiotics to prevent necrotic enteritis is under experimental breeding (Jayaraman et al. 2013; Latorre et al. 2015). Related studies have shown that *Lactobacillus* spp. can affect the absorption of dietary nutrition by enhancing the intestinal enzymes activity (Vand et al. 2014). *Lactobacillus* and *Bifidobacterium* can enhance the cellular immune function by promoting the growth of anaerobic gram-positive bacteria (Jan-Peter and Britton 2012), inhibiting the growth of gram-negative bacteria (Zuo et al. 2016), and withstand the antibiotic-associated diarrhoea and *Clostridium difficile* diarrhea (Allen et al. 2013). Furthermore, *Streptococcus* spp. and *Enterobacteriaceae* are considered as major opportunistic pathogens which could result in many diseases (Munita et al. 2012). In this research, BS15 addition significantly increased the abundance of *Lactobacillus* spp. and decreased the levels of *Streptococcus* spp. and *Enterobacteriaceae* in the jejunum and ileum, which indicated the great potential of BS15 in maintaining the balance of intestinal flora by increasing the abundance of beneficial bacteria and suppressing the colonization of harmful bacteria.

Some *Clostridium*, as the barrier in the pathogen invasion, can produce short-chain fatty acids to reduce the intestinal pH value and suppress the invasion of harmful bacteria. *C. cluster IV* and *C. cluster XIVa* are both gram-positive bacterium, which are the primary bacteria producing the butyric acid in gut (Valdés et al. 2013). *C. cluster XIVa* has the priority adhesion ability of mucoprotein and reduces the utilization of mucoprotein by intestinal pathogenic bacteria (Van et al. 2012). In this study, the contents of *C. cluster XIVa* in jejunum and ileum and *C. cluster IV* in ileum were prominently lower in the SNE model group compared with the NC group, but the BS15 reversed these changes caused by SNE, demonstrating that the addition of BS15 enhanced the intestinal barrier function in SNE chicks. Besides, the abundance of *C. cluster I* in the jejunum of SNE group was higher than that in the control group, this result was probably attributed to the augment in count of *C. perfringens* (Ignacio et al. 2015).

In conclusion, our findings yield clues to the mechanistic basis of *L. johnsonii* BS15 that may promote the growth performance and lower fat deposit in subclinical necrotic enteritis broilers, which may contribute to the improved lipid metabolism in liver and adipose tissue as well as better intestinal development, enhanced digestive ability and balanced microflora in small intestines.

## Abbreviations

SNE: subclinical necrotic enteritis
BS15: *Lactobacillus johnsonii* BS15
MRS: Man Rogosa Sharpe
ADFI: average daily feed intake
ADG: average daily gain
FCR: feed conversion rate
qRT-PCR: quantitative real-time polymerase chain reaction
ALT: alanine aminotransferase
AST: aspartate transaminase
TG: triglyceride
TC: total cholesterol
HDL-C: high-density lipoprotein cholesterol
LDL-C: low-density lipoprotein cholesterol
EGF: epidermal growth factor
IGF-1: insulin-like growth factors-1
GADPH: glyceraldehyde-3-phosphate dehydrogenase
ACC: acetyl-CoA carboxylase
FAS: fatty acid synthase
SCD1: desaturase-1
SREBP-1c: sterol regulatory element binding protein-1c
PPARα: peroxisome proliferator activated receptor α
CPT-1: carnitine palmitoyltransferase-1
ACOX: acyl CoA oxidase
PPARγ: peroxisome proliferator activated receptor γ
ATGL: adipose triglyceride lipase
LPL: lipoprotein lipase

## References

[CR1] Afrouziyeh M, Hanifian S, Taghinejad M (2014). Effects of mannan oligosaccharides on ileal digestibility of nutrients and microbial populations in the ceca of broiler chickens. Int J Biosci.

[CR2] Allen SJ, Wareham K, Wang D, Bradley C, Hutchings H, Harris W, Dhar A, Brown H, Foden A, Gravenor MB, Mack D (2013). *Lactobacilli* and *bifidobacteria* in the prevention of antibiotic-associated diarrhoea and *Clostridium difficile* diarrhoea in older inpatients (PLACIDE): a randomised, double-blind, placebo-controlled, multicentre trial. Lancet.

[CR3] Bartosch S, Fite A, Macfarlane GT, Mcmurdo ME (2004). Characterization of bacterial communities in feces from healthy elderly volunteers and hospitalized elderly patients by using real-time PCR and effects of antibiotic treatment on the fecal microbiota. Appl Environ Microbiol.

[CR4] Brown K, Decoffe D, Molcan E, Gibson DL (2012). Diet-induced dysbiosis of the intestinal microbiota and the effects on immunity and disease. Nutrients.

[CR5] Carobene A, Braga F, Roraas T, Sandberg S, Bartlett WA (2013). A systematic review of data on biological variation for alanine aminotransferase, aspartate aminotransferase and γ-glutamyl transferase. Clin Chem Lab Med.

[CR6] Chan LK, Chiu YT, Sze KM, Ng IO (2015). Tensin4 is up-regulated by EGF-induced ERK1/2 activity and promotes cell proliferation and migration in hepatocellular carcinoma. Oncotarget.

[CR7] Chávez-Tapia NC, González-Rodríguez L, Jeong MS, López-Ramírez Y, Barbero-Becerra V, Juárez-Hernández E, Romero-Flores JL, Arrese M, Méndez-Sánchez N, Uribe M (2015). Current evidence on the use of probiotics in liver diseases. J Funct Foods.

[CR8] Chen T, Gao J, Xiang P, Chen Y, Ji J, Xie P, Wu H, Xiao W, Wei Y, Wang S, Lan L, Ji H, Yan T (2015). Protective effect of platycodin D on liver injury in alloxan-induced diabetic mice via regulation of Treg/Th17 balance. Int Immunopharmacol.

[CR9] Chiva M, Soriano G, Rochat I, Peralta C, Rochat F, Llovet T, Mirelis B, Schiffrin EJ, Guarner C, Balanzó J (2002). Effect of *Lactobacillus johnsonii* La1 and antioxidants on intestinal flora and bacterial translocation in rats with experimental cirrhosis. J Hepatol.

[CR10] Compare D, Coccoli P, Rocco A, Nardone OM, De Maria S, Cartenì M, Nardone G (2012). Gut–liver axis: the impact of gut microbiota on non-alcoholic fatty liver disease. Nutr Metab Cardiovasc Dis.

[CR11] Cooper KK, Bueschel DM, Songer JG (2013). Presence of *Clostridium perfringens* in retail chicken livers. Anaerobe.

[CR12] Cui C, Shen CJ, Jia G, Wang KN (2013). Effect of dietary *Bacillus subtilis* on proportion of *Bacteroidetes* and *Firmicutes* in swine intestine and lipid metabolism. Genet Mol Res.

[CR13] Davies BS, Beigneux AP, Fong LG, Young SG (2012). New wrinkles in lipoprotein lipase biology. Curr Opin Lipidol.

[CR14] Endo H, Niioka M, Kobayashi N, Tanaka M, Watanabe T (2013). Butyrate-producing probiotics reduce nonalcoholic fatty liver disease progression in rats: new insight into the probiotics for the gut–liver axis. PLoS ONE.

[CR15] Fallah R, Kiani A, Azarfar A (2013). A review of the role of five kinds of alternatives to infeed antibiotics in broiler production. J Vet Med Anim Health.

[CR16] Fouad AM, Elsenousey HK (2014). Nutritional factors affecting abdominal fat deposition in poultry: a review. Asian-Australas J Anim Sci.

[CR17] Franks AH, Harmsen HJM, Raangs GC, Jansen GJ, Schut F, Welling GW (1998). Variations of bacterial populations in human feces measured by fluorescent in situ hybridization with group-specific 16S rRNA-targeted oligonucleotide probes. Appl Environ Microbiol.

[CR18] Goh GB, Pagadala MR, Dasarathy J, Unalparida A, Pai RK, Yerian L, Khiyami A, Sourianarayanane A, Sargent R, Hawkins C, Dasarathy S, McCullough AJ (2015). Age impacts ability of aspartate–alanine aminotransferase ratio to predict advanced fibrosis in nonalcoholic fatty liver disease. Dig Dis Sci.

[CR19] Guo X, Xia X, Tang R, Zhou J, Zhao H, Wang K (2008). Development of a real-time PCR method for Firmicutes and Bacteroidetes in faeces and its application to quantify intestinal population of obese and lean pigs. Lett Appl Microbiol.

[CR20] Ha JH, Jang J, Chung SI, Yoon Y (2016). AMPK and SREBP-1c mediate the anti-adipogenic effect of β-hydroxyisovalerylshikonin. Int J Mol Med.

[CR21] Haemmerle G, Lass A, Zimmermann R, Gorkiewicz G, Meyer C, Rozman J, Heldmaier G, Maier R, Theussl C, Eder S, Kratky D, Wagner EF, Klingenspor M, Hoefler G, Zechner R (2006). Defective lipolysis and altered energy metabolism in mice lacking adipose triglyceride lipase. Science.

[CR22] Heckmann BL, Zhang X, Saarinen AM, Liu J (2016). Regulation of G0/G1 switch gene 2 (G0S2) protein ubiquitination and stability by triglyceride accumulation and ATGL interaction. PLoS ONE.

[CR23] Ignacio A, Fernandes MR, Rodrigues VA, Groppo FC, Cardoso AL, Avilacampos MJ, Nakano V (2015). Correlation between body mass index and fecal microbiota from children. Clin Microbiol Infect.

[CR24] Jan-Peter VP, Britton RA (2012). High efficiency recombineering in lactic acid bacteria. Nucleic Acids Res.

[CR25] Jansen EH (2014). Long term stability of parameters of lipid metabolism in frozen human serum: triglycerides, free fatty acids, total-, HDL- and LDL-cholesterol, apolipoprotein-A1 and B. J Mol Biomark Diagn.

[CR26] Jayaraman S, Thangavel G, Kurian H, Mani R, Mukkalil R, Chirakkal H (2013). *Bacillus subtilis* PB6 improves intestinal health of broiler chickens challenged with *Clostridium perfringens*-induced necrotic enteritis. Poult Sci.

[CR27] Kanaki M, Kardassis D (2017). Regulation of the human lipoprotein lipase gene by the forkhead box transcription factor FOXA2/HNF-3β in hepatic cells. Biochim Biophys Acta.

[CR28] Kim YJ, Bostami ABMR, Islam MM, Hong SM, Ko SY, Yang CJ (2016). Effect of fermented ginkgo biloba and camelia sinensis-based probiotics on growth performance, immunity and caecal microbiology in broilers. Int J Poult Sci.

[CR29] Lan Y, Verstegen M, Tamminga S, Williams B (2005). The role of the commensal gut microbial community in broiler chickens. Worlds Poult Sci J.

[CR30] Latorre JD, Hernandez-Velasco X, Kuttappan VA, Wolfenden RE, Vicente JL, Wolfenden AD, Bielke LR, Prado-Rebolledo OF, Morales E, Hargis BM, Tellez G (2015). Selection of *Bacillus* spp. for cellulase and xylanase production as direct-fed microbials to reduce digesta viscosity and *Clostridium perfringens* proliferation using an in vitro digestive model in different poultry diets. Front Vet Sci.

[CR31] Layton SL, Hernandezvelasco X, Chaitanya S, Xavier J, Menconi A, Latorre JD, Kallapura G, Kuttappan VA, Wolfenden RE, Filho RLA, Hargis BM, Téllez G (2013). The effect of a-based probiotic for the control of necrotic enteritis in broilers. Food Nutr Sci.

[CR32] Lee JS, Cheng H, Damte D, Lee SJ, Kim JC, Rhee MH, Suh JW, Park SC (2013). Effects of dietary supplementation of *Lactobacillus pentosus* PL11 on the growth performance, immune and antioxidant systems of Japanese eel *Anguilla japonica* challenged with *Edwardsiella tarda*. Fish Shellfish Immunol.

[CR33] Lefterova MI, Haakonsson AK, Lazar MA, Mandrup S (2014). PPARγ and the global map of adipogenesis and beyond. Trends Endocrinol Metab.

[CR34] Ley RE, Turnbaugh PJ, Klein S, Gordon JI (2006). Microbial ecology: human gut microbes associated with obesity. Nature.

[CR35] Li X, Wang L, Li Y, Ho Y, Yang D, Chen Y, Hu X, Xue M (2011). Polysorbates as novel lipid-modulating candidates for reducing serum total cholesterol and low-density lipoprotein levels in hyperlipidemic C57BL/6J mice and rats. Eur J Pharmacol.

[CR36] Liang HW, Wang N, Wang Y, Wang F, Fu Z, Yan X, Zhu H, Diao W, Ding Y, Chen X, Zhang CY, Zen K (2015). Hepatitis B virus-human chimeric transcript HBx-LINE1 promotes hepatic injury via sequestering cellular microRNA-122. J Hepatol.

[CR37] Liu L, Fu C, Yan M, Xie H, Li S, Yu Q, He S, He J (2016). Resveratrol modulates intestinal morphology and HSP70/90, NF-κB and EGF expression in the jejunal mucosa of black-boned chickens on exposure to circular heat stress. Food Funct.

[CR38] Liu L, Ni X, Zeng D, Wang H, Jing B, Yin Z, Pan K (2016). Effect of a dietary probiotic, *Lactobacillus johnsonii* BS15, on growth performance, quality traits, antioxidant ability, and nutritional and flavour substances of chicken meat. Anim Prod Sci.

[CR39] Makawy AE (2014) Evaluation of the potential genotoxicity of antibiotics alternative probiotics used in livestock and poultry. J Food Agric Environ 12(2):389-396. https://www.researchgate.net/publication/261793725_Evaluation_of_the_potential_genotoxicity_of_antibiotics_alternative_probiotics_used_in_livestock_and_poultry

[CR40] Mandrup S, Lane MD (1997). Regulating adipogenesis. J Biol Chem.

[CR41] Matsuki T, Watanabe K, Fujimoto J, Miyamoto Y, Takada T, Matsumoto K, Tanaka R (2002). Development of 16S rRNA-gene-targeted group-specific primers for the detection and identification of predominant bacteria in human feces. Appl Environ.

[CR42] Matsuki T, Watanabe K, Fujimoto J, Takada T, Tanaka R (2004). Use of 16S rRNA gene-targeted group-specific primers for real-time PCR analysis of predominant bacteria in human feces. Appl Environ.

[CR43] Mayoral R, Osborn O, Mcnelis J, Johnson AM, Oh DY, Izquierdo CL, Chung H, Li P, Traves PG, Bandyopadhyay G, Pessentheiner A, Ofrecio JM, Cook JR, Qiang L, Accili D, Olefsky JM (2015). Adipocyte SIRT1 knockout promotes PPARγ activity, adipogenesis and insulin sensitivity in chronic-HFD and obesity. Mol Metab.

[CR44] Moosavinasab F, Ghalehkand JG, Ghanbari O, Hassanpour S (2015). In ovo injection of IGF1 improves intestinal enzyme activity in broilers. Archiv Fur Geflugelkunde.

[CR45] Munita JM, Arias CA, Murray BE (2012). Enterococcal endocarditis: can we win the war?. Curr Infect Dis Rep.

[CR46] Poudyal H, Brown L (2011). Stearoyl-CoA desaturase: a vital checkpoint in the development and progression of obesity. Endocr Metab Immune Disord Drug Targets.

[CR47] Rinttilä T, Kassinen A, Malinen E, Krogius L, Palva A (2004). Development of an extensive set of 16S rDNA-targeted primers for quantification of pathogenic and indigenous bacteria in faecal samples by real-time PCR. J Appl Microbiol.

[CR48] Saintcyr MJ, Haddad N, Taminiau B, Poezevara T, Quesne S, Amelot M, Daube G, Chemaly M, Dousset X, Guyardnicodème M (2017). Use of the potential probiotic strain *Lactobacillus salivarius* SMXD51 to control *Campylobacter jejuni* in broilers. Int J Food Microbiol.

[CR49] Sánchez B, Hevia A, González S, Margolles A (2015). Interaction of intestinal microorganisms with the human host in the framework of autoimmune diseases. Front Immunol.

[CR50] Saneyasu T, Shiragaki M, Nakanishi K, Kamisoyama H, Honda K (2013). Effects of short term fasting on the expression of genes involved in lipid metabolism in chicks. Comp Biochem Physiol B Biochem Mol Biol.

[CR51] Sheng MH, Lau KH, Baylink DJ (2014). Role of osteocyte-derived insulin-like growth factor I in developmental growth, modeling, remodeling, and regeneration of the bone. J Bone Metab.

[CR52] Shori AB (2015). The effect of encapsulating materials on the survival of probiotics during intestinal digestion: a review. Cienc E Tecnol Dos Mater.

[CR53] Song S, Attia RR, Connaughton S, Niesen MI, Ness GC, Elam MB, Hori RT, Cook GA, Park EA (2010). Peroxisome proliferator activated receptor α (PPARα) and PPAR gamma coactivator (PGC-1α) induce carnitine palmitoyltransferase IA (CPT-1A) via independent gene elements. Mol Cell Endocrinol.

[CR54] Suda Y, Villena J, Takahashi Y, Hosoya S, Tomosada Y, Tsukida K, Shimazu T, Aso H, Tohno M, Ishida M, Makino S, Ikegami S, Kitazawa H (2014). Immunobiotic *Lactobacillus jensenii* as immune-health promoting factor to improve growth performance and productivity in post-weaning pigs. BMC Immunol.

[CR55] Sun H, Ni X, Song X, Wen B, Zhou Y, Zou F, Yang M, Peng Z, Zhu H, Zeng Y, Wang H, Fu X, Shi Y, Yin Z, Pan K, Jing B, Zeng D, Wang P (2016). Fermented *Yupingfeng* polysaccharides enhance immunity by improving the foregut microflora and intestinal barrier in weaning rex rabbits. Appl Microbiol Biotechnol.

[CR56] Tanos R, Murray IA, Smith PB, Patterson A, Perdew GH (2012). Role of the Ah receptor in homeostatic control of fatty acid synthesis in the liver. Toxicol Sci.

[CR57] Tsiouris VV (2016). Poultry management: a useful tool for the control of necrotic enteritis in poultry. Avian Pathol.

[CR58] Valdés L, Estévez PG, Salazar N, Rios-Covián D, González-Muñoz MJ, Parajó JC, Ruas-Madiedo P, Gueimonde M, Reyes-Gavilán CGDL (2013). Population dynamics of some relevant intestinal microbial groups in human fecal batch cultures with added fermentable xylooligosaccharides obtained from rice husks. BioResources.

[CR59] Van AP, Belzer C, Goossens M, Kleerebezem M, De Vos WM, Thas O, De WR, Kerckhof FM, Van de WT (2012). Butyrate-producing Clostridium cluster XIVa species specifically colonize mucins in an in vitro gut model. ISME J.

[CR60] Vand ZDA, Alishahi M, Tabande MR (2014). Effects of different levels of *Lactobacillus casei* as probiotic on growth performance and digestive enzymes activity of *Barbus gryprus*. Int J Biosci.

[CR61] Walter J, Hertel C, Tannock GW, Lis CM, Munro K, Hammes WP (2001). Detection of *Lactobacillus*, *Pediococcus*, *Leuconostoc*, and *Weissella* species in human feces by using group-specific PCR primers and denaturing gradient gel electrophoresis. Appl Environ.

[CR62] Wang YH, Huang Y (2014). Effect of *Lactobacillus acidophilus* and *Bifidobacterium bifidum* supplementation to standard triple therapy on *Helicobacter pylori* eradication and dynamic changes in intestinal flora. World J Microbiol Biotechnol.

[CR63] Wang H, Ni X, Lei L, Dong Z, Jing L, Qing X, Li G, Pan K, Bo J (2017). Controlling of growth performance, lipid deposits and fatty acid composition of chicken meat through a probiotic, *Lactobacillus johnsonii* during subclinical *Clostridium perfringens* infection. Lipids Health Dis.

[CR64] Xin J, Zeng D, Wang H, Ni X, Yi D, Pan K, Jing B (2014). Preventing non-alcoholic fatty liver disease through *Lactobacillus johnsonii* BS15 by attenuating inflammation and mitochondrial injury and improving gut environment in obese mice. Appl Microbiol Biotechnol.

[CR65] Xu S, Wang D, Zhang P, Lin Y, Fang Z, Che L, Wu D (2015). Oral administration of *Lactococcus lactis*-expressed recombinant porcine epidermal growth factor (rpEGF) stimulates the development and promotes the health of small intestines in early-weaned piglets. J Appl Microbiol.

[CR66] Yang KT, Lin C, Liu CW, Chen YC (2014). Effects of chicken-liver hydrolysates on lipid metabolism in a high-fat diet. Food Chem.

[CR67] Yu JH, Lee YJ, Kim HJ, Choi H, Choi Y, Seok JW, Kim JW (2015). Monoacylglycerol *O*-acyltransferase 1 is regulated by peroxisome proliferator-activated receptor γ in human hepatocytes and increases lipid accumulation. Biochem Biophys Res Commun.

[CR68] Zhou X, Wang Y, Gu Q, Li W (2010). Effect of dietary probiotic, *Bacillus coagulans*, on growth performance, chemical composition, and meat quality of *Guangxi yellow* chicken. Poult Sci.

[CR69] Zhou M, Zeng D, Ni X, Tu T, Yin Z, Pan K, Jing B (2016). Effects of *Bacillus licheniformis* on the growth performance and expression of lipid metabolism-related genes in broiler chickens challenged with *Clostridium perfringens*-induced necrotic enteritis. Lipids Health Dis.

[CR70] Zhuang S, Jiang FB, Jia ZX, Yan R (2015) *Clostridium butyricum* can be used as a potential alternative for the antibiotic in Cherry Valley ducks. J Anim Plant Sci 25(5):1227-1232. http://www.thejaps.org.pk/docs/v-25-05/04.pdf

[CR71] Zuo F, Yu R, Feng X, Chen L, Zeng Z, Khaskheli GB, Ma H, Chen S (2016). Characterization and in vitro properties of potential probiotic *Bifidobacterium* strains isolated from breast-fed infant feces. Ann Microbiol.

